# Mathematical Models for a New Era of Malaria Eradication

**DOI:** 10.1371/journal.pmed.0050231

**Published:** 2008-11-25

**Authors:** Maciej F Boni, Caroline O Buckee, Nicholas J White

## Abstract

Maciej Boni and colleagues discuss a new model exploring how a switch in antimalarial drug use to artemisinin-based combination therapies will affect malaria prevalence and incidence in endemic regions.

The renewed focus on malaria control and eradication in recent years has shifted the design of malaria control programs away from short-term, local solutions towards more wide-ranging and long-term strategies. This new era of malaria control will require sustained commitment and funding, with proposed control programs being evaluated in terms of geographic scope and long-term feasibility [1]. Because this type of evaluation cannot be done experimentally, mathematical modeling has emerged as a popular tool for comparing the possible strategies for the control and elimination of malaria.

Historically, the mathematical models of Ross, Macdonald, and Dietz gave us insights into the power of certain malaria control strategies, such as targeting female anopheline mosquitoes and using integrated approaches for malaria control [2]. These and other early modeling efforts [3–5] contributed to policy and provoked widespread discussion and new theoretical and experimental work. Early models usually had simple designs aimed at understanding the basic principles of the parasites' population biology and evolution. These models gave us a threshold principle in mosquito control, which states that mosquito numbers need only to be reduced below a certain threshold in order to set the parasites on a path to extinction, as well as combination therapies as a means of delaying resistance evolution. With increasing computing power over the past decades, mathematical models have become more detailed and complex. Recently, the largest malaria modeling project known to date was funded by the Bill & Melinda Gates Foundation and uses a network of volunteer computers to run many variations of a highly complex malaria model [6]. While these complex models can make specific, quantitative predictions about strategies for control, simple models are often more appropriate for deriving general principles about malaria epidemiology. Both approaches will be important for developing coordinated malaria control policies.

**Linked Research Article**

This Perspective discusses the following new study published in *PLoS Medicine*:

Okell LC, Drakeley CJ, Bousema T, Whitty CJM, Ghani AC (2008) Modelling the impact of artemisinin combination therapy and long-acting treatments on malaria transmission intensity. PLoS Med 5(11): e226. doi:10.1371/journal.pmed.0050226

Lucy Okell and colleagues predict the impact on transmission outcomes of ACT as first-line treatment for uncomplicated malaria in six areas of varying transmission intensity in Tanzania.

## Effects of Antimalarials on Transmission and Incidence

In this issue of *PLoS Medicine*, Lucy Okell and colleagues present a mathematical model exploring how a switch in antimalarial drug use to artemisinin-based combination therapies (ACT) will affect malaria prevalence and incidence in endemic regions [7]. ACTs, increasingly used as a first-line treatment for uncomplicated malaria, work rapidly and act on the transmission stages of the parasite. Their role in reducing transmission makes them a potentially important component of future malaria elimination and eradication efforts. Theoretical studies of the kind presented by Okell and colleagues provide valuable insights into how best to use ACTs in different regions.

Using data from a large cross-sectional survey of individuals from six different transmission settings in Tanzania to estimate the parameter inputs of the model, Okell and colleagues compare the effects of ACT introduction in areas of high and low malaria transmission. The model is somewhat complex (as indeed are malaria infections), but this complexity is necessary to include some important details about disease progression and superinfection. The model is simple enough, however, that a careful reading of the supplementary materials suffices for the interested reader to understand its core components and mechanisms. It is also simple enough that it can be run repeatedly on a personal computer to assess how changes in the model assumptions would affect the results.

One general prediction of the model with potentially important policy implications is that gametocytocidal drugs, such as artemisinins and 8-aminoquinolines, may reduce malaria transmission more effectively in low-transmission settings, while drugs with longer prophylactic effects might have a bigger impact in high-transmission settings where biting is much more frequent. This result can be tested in more detail via independently built models working under different assumptions and using data coming in from the field. If true, the result would provide guidance for drug choice in local malaria control. The implications are that control programs in high-transmission areas should use slowly eliminated antimalarials and other control measures to lower transmission, and then introduce specific gametocytocidal drugs as these measures take effect and transmission intensity falls. This result does not take into account the potential for drug-resistance evolution.

An important quantitative prediction of the model is that prevalence reductions in the Tanzania study can only be as high as 50%, even with 100% ACT coverage. Another prediction is that this maximum prevalence reduction would be lower in high-transmission areas where most parasites live in asymptomatic individuals. Asymptomatic carriage, transmission before treatment, and dormancy (hypnozoites) in P. vivax and P. ovale are the principal reasons why improving access to drug treatments alone may not eliminate malaria (although improving ACT coverage alone has had some notable and well documented successes [8]). Fortunately, we will not be relying on drugs alone in our control efforts; increased drug availability will be combined with distribution of free long-lasting insecticide-treated bednets and, where appropriate, indoor residual spraying and other methods of vector control (and even, perhaps, a vaccine). Mathematical modeling will continue to provide insights into the best ways to combine these different approaches to malaria control.

## Role of Mathematical Models in Containing Drug Resistance

We predict an increase in malaria mathematical modeling and a danger of increased confusion among policy makers as each investigation strives for a novel result of “interest.” Malaria models predictably generate heat, but less often light. The keys to a sound and understandable modeling conclusion are appropriate design, working within the model's assumptions, a careful analysis of the model's sensitivity to these assumptions, and a clear statement of the model's limitations. Following these basic precepts will make modeling accessible to the medical and public health communities, who need to trust and understand the modelers' recommendations. In predicting the effects of antimalarial drugs on malaria incidence and prevalence, we have to be clear that our estimates of transmission dynamics, and in particular the effects of antimalarial drugs on malaria transmission and their interaction with host immunity, are poor. Research on this critical area has not kept up with developments in other areas of malaria research.

With the caveats above in mind, Okell and colleagues provide us with a useful predictive model, which gives a picture of short-term results in Tanzania under the ideal condition of 100% ACT coverage. Realistically, though, ACT coverage following introduction in endemic regions might be 30%–60%, especially in the short term [9]. Any long-term policy with a high coverage goal should have inbuilt mechanisms capable of responding to the rapid evolution of artemisinin resistance, particularly since there is currently no adequate pharmacological replacement for the artemisinin-class drugs. Beyond the general principle of combination therapy, there is no consensus on how to respond to or prevent the emergence and spread of drug resistance in the long term, although this is an active area of research [10–13]. If we can secure sustained adequate funding, and overcome all the political and operational obstacles, then the evolution of mosquito resistance to current insecticides and parasite resistance to current ACTs are the greatest dangers we face in our present attempts to control malaria [14]. Mathematical modeling is an important tool for developing strategies to contain the threat of resistance.

In the end, whether the goal is control, elimination, or eradication, a broad and sustainable plan is necessary to ensure that disease reductions are achieved and maintained. In the absence of such large-scale long-term planning, we risk the emergence of widespread antimalarial drug resistance, and in particular loss of the artemisinins, endangering the health of hundreds of millions of people who rely on these drugs as their primary malaria treatment [15]. Our planning and modeling efforts should focus on preserving the efficacy of our current drugs and insecticides as the minimum requirement for any chance of success in the control and eventual eradication of malaria.

## Abbreviations

ACT: artemisinin-based combination therapy
